# Ceftriaxone-Induced Pancytopenia: A Case Report

**DOI:** 10.3390/hematolrep17030030

**Published:** 2025-06-12

**Authors:** Edin Karisik, Zorica Stanojevic-Ristic, Marija Jevtic, Julijana Rasic, Miljana Maric, Milica Popovic

**Affiliations:** 1 Department of Internal Medicine, General Hospital Novi Pazar, 36300 Novi Pazar, Serbia; 2 Department of Pharmacology and Toxicology, Faculty of Medicine, University of Pristina, 38220 Kosovska Mitrovica, Serbia; zorica.stanojevic@med.pr.ac.rs (Z.S.-R.); julijana.rasic@med.pr.ac.rs (J.R.); 3 Department of Pediatrics, Health Center Kosovska Mitrovica, 38220 Kosovska Mitrovica, Serbia; marija.jevtic91@outlook.com; 4 Emergency Center of Serbia, Department of Neuroradiology, 11000 Belgrade, Serbia; miljanaa_np@yahoo.com; 5 Clinical and Hospital Center Gračanica, Pediatric Clinic, 38205 Gracanica, Serbia; milicavasicpopovic@gmail.com

**Keywords:** pancytopenia, ceftriaxone, idiosyncrasy, filgrastim, antibiotics

## Abstract

**Background:** Cephalosporins are considered safe antibiotics. However, serious hematological abnormalities may occur, although rarely, after their therapeutic use. **Case Presentation:** We present a case of pancytopenia in a 72-year-old female patient treated with ceftriaxone for a urinary tract infection. After five days of therapy, pancytopenia was observed. Other causes were excluded through extensive diagnostic evaluation, including immunological tests, viral serologies, bone marrow aspiration, and peripheral blood smear. The patient’s clinical condition significantly improved following the discontinuation of ceftriaxone and the administration of granulocyte colony-stimulating factor (G-CSF). Bone marrow findings revealed hypocellularity without malignant infiltration, and peripheral smear showed no dysplasia, blasts, or hemolysis. **Conclusions:** This case demonstrates that ceftriaxone, although widely regarded as a safe antibiotic, can induce rare but serious hematologic complications such as pancytopenia. A high index of suspicion is required when patients on antibiotic therapy develop unexplained cytopenias. Detailed medication history, exclusion of other causes, and prompt discontinuation of the suspected drug are essential. The patient’s favorable outcome supports the likelihood of an idiosyncratic, immune-mediated mechanism. Future research should explore pharmacogenomic screening in patients at increased risk, particularly involving HLA variants.

## 1. Introduction

Pancytopenia is characterized by a simultaneous reduction in red blood cells, white blood cells, and platelets in the peripheral blood, and it can be caused by infections, hematologic malignancies, autoimmune disorders, and drugs [1]. Among medications, hematologic reactions are more commonly associated with chemotherapy agents but can also occur with antibiotics such as cephalosporins [2,3].

Cephalosporins are β-lactam antibiotics classified into five generations based on their spectrum of coverage against Gram-positive and Gram-negative bacteria, resistance to beta-lactamases, pharmacokinetics, and their temporal discovery. They are generally considered safe and well-tolerated antibiotics [4].

Ceftriaxone, a third-generation cephalosporin, is widely used as a parenteral antibacterial to treat a variety of infections [4]. Its extended coverage of Gram-negative bacteria, favorable tissue penetration for most common infections, low allergenic and toxic profile, and once-daily dosing make it a very popular drug for both outpatient and inpatient antimicrobial therapy [5,6]. However, hematologic complications, including neutropenia and thrombocytopenia, have been reported [7,8]. Hematologic toxicity associated with ceftriaxone is rare, but serious potential complications can occur after standard doses and usual duration of therapy with this drug [9]. The pathogenesis of this toxicity is not fully explained, but it has been suggested to occur either by an immunologic mechanism or because of direct drug toxicity [8]. The hematological toxicity of ceftriaxone has been described in case studies and those involving special patient groups [8,10].

This case report examines a 72-year-old female patient treated with ceftriaxone. The aim is to highlight the complexity of a rare but serious hematological adverse reaction associated with ceftriaxone use—pancytopenia. It underscores the importance of clinical vigilance, timely recognition, and appropriate management in preventing severe outcomes.

## 2. Case Presentation

A 72-year-old female was hospitalized for fever and general weakness. Although the patient was being treated for a urinary tract infection, she showed no signs of sepsis. Both C-reactive protein (CRP) and procalcitonin (PCT) levels were only mildly elevated, and there was no clinical or laboratory evidence of systemic inflammatory response syndrome (SIRS) or organ dysfunction. In her personal medical history, the patient reported being aware of a previously diagnosed Baker’s cyst but denied any other chronic illnesses or known allergies to food or medications. She was not on any chronic therapy or supplements but reported occasional use of paracetamol for musculoskeletal pain relief. There was no relevant family history, including autoimmune or hematological diseases. She was receiving treatment for a urinary tract infection with ceftriaxone (2 g/day intravenously). After five days of therapy, laboratory findings revealed a significant decline across all blood cell lines (Table 1).

There was no evidence of hemolysis, bleeding, ongoing infection, or infiltrative bone marrow disease. During hospitalization, the patient received prophylactic subcutaneous fraxiparine due to detected superficial venous thrombosis. Given the thrombocytopenia, heparin-induced thrombocytopenia (HIT) was considered in the differential diagnosis. However, the 4T score was low < 5%, and there were no clinical or laboratory findings consistent with HIT. Pancytopenia was therefore attributed to ceftriaxone as the most likely cause. Immunological investigations, viral serologies, and bone marrow aspiration excluded alternative causes. A peripheral blood smear was also performed and revealed no evidence of hemolysis, dysplasia, or blasts, further supporting a nonmalignant etiology.

Radiologic evaluation was performed during hospitalization. Doppler ultrasound of the lower extremities revealed a thrombotic mass in the left great saphenous vein without involvement of the deep venous system. Soft tissue ultrasound showed a hypoechoic lesion in the left lower leg without signs of abscess formation, along with subcutaneous edema and a Baker’s cyst in the popliteal region. Abdominal ultrasound revealed a 20 mm hemangioma in the left liver lobe, and pelvic Doppler ultrasound showed a small amount of free fluid without lymphadenopathy. Standard radiographs confirmed degenerative bone changes in the upper abdomen and pelvis.

**Figure 1:**
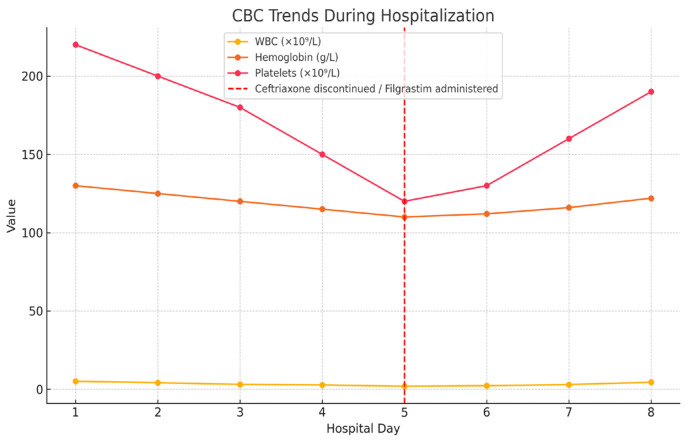
Trends in white blood cell count (WBC), hemoglobin, and platelet levels during hospitalization. Ceftriaxone was discontinued and filgrastim was administered on day 5 (indicated by red dashed line).

Ceftriaxone was discontinued, and filgrastim was administered subcutaneously at a dose of 30 MU (approximately 300 mcg) on day 5 of hospitalization. According to the medication administration chart, only a single dose of filgrastim was given. Trends in hematologic parameters throughout hospitalization are shown in Figure 1.

## 3. Discussion

Drug-induced hematologic reactions can arise from direct toxic effects on hematopoiesis or immune-mediated idiosyncratic mechanisms [11,12]. Idiosyncratic drug reactions are unpredictable, dose-independent, and often immune-mediated [13,14]. These reactions can be triggered by a variety of medications, including antibiotics, and their onset is often difficult to predict. The underlying mechanisms behind such reactions are complex and not fully understood, but they are believed to involve immune-mediated processes that lead to hematologic dysfunction.

In this case, the clinical timeline, reversibility after drug withdrawal, and favorable response to granulocyte colony-stimulating factor (G-CSF) strongly suggest an idiosyncratic mechanism [8,11]. The patient exhibited a clear temporal relationship between ceftriaxone administration and the development of pancytopenia, which improved rapidly upon the discontinuation of the drug and the introduction of G-CSF. This supports the idea that the hematologic reaction was likely drug-induced and immune-mediated, rather than resulting from a direct toxic effect on the bone marrow.

Although cephalosporins are generally considered safe, serious hematologic adverse effects such as neutropenia and pancytopenia have been reported, particularly in susceptible individuals [3,15]. These reactions are rare, but they can be severe and life-threatening, highlighting the importance of monitoring patients closely during antibiotic therapy, especially when other risk factors are present. The exact mechanism by which cephalosporins induce hematologic toxicity is still unclear, but it may involve immune-mediated destruction of blood cells.

One of the key mechanisms through which ceftriaxone may induce pancytopenia is through immune-mediated processes. Cephalosporins, including ceftriaxone, can trigger the formation of antigens on the surface of blood cells, leading to the production of antibodies that target and destroy these cells. This phenomenon is known as the hapten reaction, where the drug, acting as a hapten, modifies surface proteins on red blood cells, platelets, or leukocytes, leading to their rapid immune-mediated clearance [16]. This process can result in various hematologic disorders, such as hemolytic anemia, neutropenia, and thrombocytopenia, ultimately culminating in pancytopenia. This mechanism is specific to ceftriaxone but can also be seen with other beta-lactam antibiotics.

Recent findings have also shown that medications beyond antibiotics, including antiepileptics and analgesics, have been implicated in drug-induced agranulocytosis and pancytopenia [17,18]. This suggests that drug-induced hematologic reactions are not limited to antibiotics and can occur with various medications [19]. Such reactions may often go underreported or overlooked, as their presentation can resemble other hematologic disorders. Therefore, a thorough evaluation and comprehensive drug history are essential for diagnosing patients with unexplained hematologic abnormalities.

Several drugs beyond antibiotics have been associated with pancytopenia through a variety of mechanisms, including direct myelotoxicity, idiosyncratic reactions, and immune-mediated suppression. Table 2 summarizes the main pharmacological classes and representative agents implicated in drug-induced pancytopenia, along with their proposed mechanisms and relevant clinical considerations.

The role of genetic and pharmacogenetic factors, particularly human leukocyte antigen (HLA) variants, has been highlighted in recent research on drug-induced hematologic reactions. Studies suggest that specific HLA variants may predispose individuals to these reactions, including those triggered by ceftriaxone [20]. The genetic makeup of the individual can significantly influence the immune response to the drug. In certain cases, the immune system may recognize modified blood cells as foreign and initiate an immune response that leads to their destruction. This highlights the importance of considering genetic factors in patients who may be at higher risk for such reactions.

While most of the data on HLA associations come from studies on clozapine-induced agranulocytosis, similar immune-mediated mechanisms are likely at play in antibiotic-induced hematologic reactions [20]. Identifying patients with genetic predispositions could help guide clinical decisions and potentially prevent severe reactions.

In recent years, the potential role of genetic predisposition in drug-induced hematologic toxicity has garnered increasing attention. In particular, certain HLA variants have been associated with increased susceptibility to severe adverse drug reactions, including agranulocytosis and aplastic anemia. For instance, HLA-B*38:02 and HLA-DRB*108:03 have been implicated in antithyroid drug-induced agranulocytosis [21], and HLA-B*57:01 is strongly associated with abacavir hypersensitivity [22].

Although no specific HLA genotype has yet been definitively linked to ceftriaxone-induced pancytopenia, immune-mediated reactions involving HLA-restricted pathways remain a plausible mechanism. As pharmacogenomic research progresses, identifying such genetic risk factors may become essential in predicting which patients are more likely to develop hematologic complications.

In this context, genetic screening could be considered in selected high-risk populations, such as individuals with a history of drug-induced cytopenias or multiple drug allergies. While routine HLA genotyping is not currently standard clinical practice in antibiotic prescribing, future integration of genetic risk assessment into clinical workflows may enhance the safety of antimicrobial therapy through personalized risk stratification.

The management of drug-induced hematologic reactions primarily involves the early identification and discontinuation of the offending agent, as well as supportive care. In more severe cases, the administration of G-CSF has been shown to promote hematologic recovery [11,23]. G-CSF can accelerate recovery in some cases, although its effectiveness may vary based on the severity of the reaction. It is critical to tailor treatment to each individual patient’s needs and their response to therapy.

The effectiveness of G-CSF in facilitating recovery has been demonstrated in several reports, including this case, where G-CSF played a crucial role in the patient’s rapid recovery, further supporting the diagnosis of drug-induced myelosuppression [20]. However, it is important to note that not all patients will respond to G-CSF, and its use should be considered on a case-by-case basis.

This case underscores the importance of vigilance when prescribing even commonly used antibiotics. While ceftriaxone and other cephalosporins are generally considered safe, it is vital for clinicians to remain alert to the potential for rare but serious adverse reactions. Early recognition and timely management of drug-induced hematologic reactions can lead to full recovery and significantly reduce the risk of complications [24]. By raising awareness of such reactions, clinicians can improve patient outcomes and prevent the potentially life-threatening consequences of drug-induced hematologic disorders.

## 4. Conclusions

This case demonstrates that ceftriaxone, although widely regarded as a safe antibiotic, can induce rare but serious hematologic complications such as pancytopenia. A high index of suspicion is required when patients on antibiotic therapy develop unexplained cytopenias. Detailed medication history, exclusion of other causes, and prompt discontinuation of the suspected drug are essential. The patient’s favorable outcome following drug withdrawal and G-CSF administration supports the likelihood of an idiosyncratic, immune-mediated mechanism. Considering emerging evidence on genetic susceptibility, particularly involving HLA variants, future research should explore pharmacogenomic screening in patients at increased risk for drug-induced hematologic toxicity.

## Figures and Tables

**Table 1:** Laboratory parameters during hospitalization and after ceftriaxone withdrawal and filgrastim administration.

Parameter	Day 1	Day 5	Day 7	After Ceftriaxone Withdrawal and Filgrastim Administration
Hematology				
White blood cells (WBC) × 10^9^/L	3.5	2.2	1.8	4.6
Red blood cells (RBC) × 10^12^/L	3.8	3.5	3.2	4.0
Hemoglobin (g/L)	112	102	98	120
Hematocrit (%)	33.6	31.2	30.0	36.1
Platelets (×10⁹/L)	130	92	88	101
Neutrophils (%)	75.2	61.2	53.0	68.0
Lymphocytes (%)	22.1	26.8	30.0	24.0
Biochemistry and inflammatory markers				
C-reactive protein (CRP) mg/L	22.4	34.2	47.4	18.3
Procalcitonin (PCT) ng/mL	0.23	0.34	0.29	0.10
AST (U/L)	31	38	42	29
ALT (U/L)	26	33	36	25
LDH (U/L)	241	270	296	212
Total bilirubin (µmol/L)	8.7	10.3	11.5	6.2
Creatinine (µmol/L)	83	91	87	79
Urea (mmol/L)	4.6	5.1	4.3	4.0
Microbiological work-up				
Hemoculture	-	-	-	Negative ×3
Urine culture	-	-	-	Negative
Tumor markers				
CEA (ng/mL)	-	2.0	-	-
AFP (ng/mL)	-	3.5	-	-
CA 19-9 (U/mL)	-	22.5	-	-
CA 15-3 (U/mL)	-	17.8	-	-
CA 125 (U/mL)	-	34.5	-	-
Cyfra 21-1 (ng/mL)	-	1.7	-	-
Immunological and viral serology				
ANA	-	-	-	Negative
ENA	-	-	-	Negative
ANCA	-	-	-	Negative
dsDNA	-	-	-	Negative
HBsAg	-	-	-	Negative
Anti-HCV	-	-	-	Negative
Anti-HIV	-	-	-	Negative
CMV IgM	-	-	-	Negative
EBV IgM	-	-	-	Negative
Toxoplasma gondii	-	-	-	Negative
Other tests				
ASTO (U/mL)	-	-	-	125
Throat and nasal swab	-	-	-	Negative
Bone marrow aspiration Peripheral blood smear	-	-	-	Hypocellular marrow, no malignant infiltration No evidence of hemolysis, dysplasia, or blasts

AST: aspartate transaminase; ALT: alanine transaminase; LDH: lactate dehydrogenase; CEA: carcinoembryonic antigen; AFP: alpha-fetoprotein; CA 19-9: cancer antigen for pancreatic cancer; CA 15-3: cancer antigen for breast cancer; CA 125: cancer antigen for ovarian cancer; Cyfra 21-1: tumor marker for squamous cell lung cancer; ANA: antinuclear antibody; ENA: extractable nuclear antigens antibodies; ANCA: anti-neutrophil cytoplasmic antibodies; dsDNA: double-stranded DNA antibodies; HBsA: hepatitis B surface antigen; Anti-HCV: anti-hepatitis C virus antibody; Anti-HIV: anti-human immunodeficiency virus antibody; CMV IgM: cytomegalovirus immunoglobulin M; EBV IgM: Epstein–Barr virus immunoglobulin M; ASTO: anti streptolysin O test.

**Table 2:** Drugs associated with pancytopenia.

Drug Class	Example(s)	Mechanism of Pancytopenia	Notes	References
Biologics and Monoclonal Abs	Rituximab, Infliximab	Immune-mediated cytopenias	Pancytopenia is rare but has been documented	[2]
Chemotherapy agents	Methotrexate, Cyclophosphamide	Dose-dependent bone marrow suppression	Expected adverse effect; supportive care needed	[2]
Immunosuppressants	Azathioprine, Mycophenolate mofetil	Inhibition of marrow cell proliferation	Requires frequent CBC monitoring	[2]
Antiretroviral drugs	Zidovudine (AZT)	Mitochondrial toxicity affecting marrow cells	Pancytopenia often dose-related	[2,3]
NSAIDs	Phenylbutazone, Indomethacin	Immune-mediated or dose-dependent suppression	Rare but severe cases have been reported	[2,3]
Antimalarials	Chloroquine, Quinine	Immune-mediated hemolysis and marrow suppression	Rare; often reversible	[3]
Antiepileptics	Carbamazepine, Phenytoin, Valproate	Direct toxicity or idiosyncratic reaction	Requires regular blood count monitoring	[2,3,11]
Antipsychotics	Clozapine	Agranulocytosis with potential for pancytopenia	Requires regular CBC monitoring	[2,3,11]
Sulfonamides	Sulfamethoxazole-trimethoprim	Idiosyncratic or immune-mediated	More common in elderly and HIV patients	[2,3,11]
Beta-lactam antibiotics	Ceftriaxone, Penicillin, Piperacillin	Immune-mediated or direct bone marrow toxicity	Usually reversible after drug withdrawal	[5,7,11,15]
Antithyroid drugs	Methimazole, Propylthiouracil	Idiosyncratic immune-mediated bone marrow suppression	Associated with agranulocytosis and pancytopenia	[8,11,18]
Antifungals	Amphotericin B	Direct bone marrow suppression	Requires close monitoring	[19]

## Data Availability

No new data were created or analyzed in this study.
